# Foreign-born women rated medical and emotional aspects of postnatal care higher than women born in Sweden: A quantitative comparative study

**DOI:** 10.18332/ejm/172573

**Published:** 2023-11-14

**Authors:** Ingegerd Hildingsson, Helene Parment, Ulrika Öhrn, Margareta Johansson

**Affiliations:** 1 Department of Women's and Children's Health, Faculty of Medicine, Uppsala University, Uppsala, Sweden; 2 Department of Nursing, Mid Sweden University, Sundsvall, Sweden; 3 Department of Nursing, Umea University, Umea, Sweden; 4 Sundsvall Regional Hospital, Sundsvall, Sweden

**Keywords:** immigrants, postnatal care, questionnaire, native born, women

## Abstract

**INTRODUCTION:**

Although high-quality postnatal care provides information and recognizes women’s personal and cultural contexts, foreign-born women are more exposed to poor health and adverse birth outcomes. The aim of this study was to compare the length and model of postnatal care, along with the content of care, between foreign-born and native-born women living in Sweden. Another aim was to explore factors associated with being very satisfied with various aspects of postnatal care.

**METHODS:**

This was a descriptive cross-sectional study of 483 postnatal women in two Swedish hospitals in 2017. Women completed a questionnaire comprising background data, pregnancy and birth related variables and the Early Postnatal Questionnaire. Data were analyzed using descriptive statistics, analysis of variance and multivariate logistic regression analyses.

**RESULTS:**

Foreign-born women were more likely to have a shorter (<24 h) or longer (>48 h) length of postnatal stay than women born in Sweden. No differences in birth outcomes emerged between the two groups. Foreign-born women rated the medical (OR=1.77; 95% CI: 1.04–3.03) and emotional (OR=2.0; 95% CI: 1.17–3.40) aspects of postnatal care as being more important than Swedish-born women did. The most important aspect of overall satisfaction was the content of care, and the subscale Caring Relationship (AOR=8.15; 95% CI: 4.87–14.62) outscored all other aspects.

**CONCLUSIONS:**

Important factors of satisfactory experiences with postnatal care in a Swedish context were receiving information, professional care, and a hospital environment that facilitates recovery after labor and birth. Culturally sensitive and individualized postnatal care with continuity should therefore be prioritized.

## INTRODUCTION

For childbearing women, the postnatal period includes a significant transition characterized by changes in self-identity, changes in roles in their relationships, and openings for personal growth. To manage the transition, women have expressed the need for practical, emotional, and psychological support from family members, peer groups, and healthcare providers such as midwives, including during the postnatal period^1^. They also want individually tailored approaches and care for their own and their infants’ needs. To be sure, high-quality postnatal care provides consistent information, the recognition of the women’s personal and cultural contexts^1^, and the continuity of care needed to facilitate trusting relationships^1,2^. Among migrant women in particular, continuity of care has been rated as an important factor in establishing trusting relationships^2,3^. Fragmented care given by different midwives, by contrast, has been found to negatively influence the effectiveness of care and women’s confidence in attending appointments^3^.

Contemporary postnatal care is characterized by short length of hospital stay and stressful environments, both of which limit midwives’ time to provide information and check-ups to prepare women for parenting^4,5^. In a large Cochrane review comprising 17 scientific studies with more than 9000 participants, the short length of postnatal hospital stays received particular focus; however, because no clear definition of early discharge and the design of the studies varied, the authors graded the evidence as being low to moderate^6^. Their results showed that early discharge might increase infants’ re-admission to hospitals within 28 days after birth but did not significantly affect women’s re-admission, the risk of depression, or the length of the breastfeeding period^6^.

Knowledge about the length of postnatal hospital stay and women’s satisfaction with postnatal care is somewhat limited. Even so, several authors have reported that women’s opinions about the length of stay are more important than the actual hours per stay^6,7^. In addition, access to professional support has been highlighted as important when women are discharged early after birth^8-10^. Moreover, migrant women in European countries during the postnatal period have reported receiving restricted support due to receiving only limited information in an accessible language or format. Among those women, those with family present in their new home countries appreciated their assistance, guidance, and support; however, many of the women lacked social support, which left them feeling lonely, isolated, and distressed. Without family support, the women had difficulties raising the children and experienced tension in their relationships with their partners^3^.

Models of postnatal care have been associated with maternal satisfaction, as shown in a randomized controlled trial in which women who received postnatal care at home reported higher satisfaction and fewer breastfeeding problems than women who received such care in clinics^11^. A systematic review of 53 studies conducted in postnatal wards in the United Kingdom reported that women were mostly satisfied with their postnatal care but less so with the environment and their interactions with staff. It was also obvious that ethnic-minority women were less satisfied with postnatal care than their non-ethnic-minority counterparts were^5^. Foreign-born women living in Sweden constitute a heterogeneous population with different experiences, backgrounds, health needs, and health-related behaviors. Moreover, foreign-born women living in Sweden are reported to experience more adverse birth outcomes^12,13^, higher levels of fear of birth^14,15^, and stillbirth^16^.

### Problem area

When it comes to foreign-born women, postnatal care is an underexamined area. Although foreign-born women are well-known to be more exposed to poor health and adverse birth outcomes, studies on experiences with postnatal care in Sweden in that population have been rare. Knowledge about the content of postnatal care and women’s satisfaction with the care given also remains limited. Against that background, the aim of this study was to compare the length and model of postnatal care as well as the content of care between foreign-born and native-born women living in Sweden. An additional aim was to explore factors associated with being very satisfied with various aspects of postnatal care regardless of women’s country of origin.

## METHODS

### Design and setting

A descriptive cross-sectional study was conducted in two hospitals in northern Sweden with annual birth rates of 1700 and 700, respectively. The study was approved by the Regional Ethics Committee (Dnr 2017-442-31).

*Context of postnatal care in the study*

The hospitals in the present study offer a traditional postnatal ward, primarily for women with complicated births or who need breastfeeding support. Women with uncomplicated births and healthy infants are encouraged to accept early discharge, namely after a minimum of 6 h and the first pediatric examination. In the largest hospital, there is a hotel ward close to the postnatal ward that some new parents use in order to rest before leaving the hospital. Because women who choose the hotel ward are discharged administratively from postnatal care and because the ward is not staffed with midwives, women are instructed to call the postnatal ward if they need help or support. In the neonatal ward, by contrast, parents have the option of co-care. Partners are usually allowed to stay overnight in the labor ward before going home, stay in the hotel ward, or, in most cases, stay in the postnatal ward. After birth, women return with their infants to the hospital within 48 h for a blood sample to detect any metabolic disorders (i.e. PKU test) and within a week for a second pediatric examination.

*Recruitment of participants*

Women who gave birth in the first 6 months of 2017 were identified (n=1226) using the electronic database at the hospitals. The database contains information about languages, and women in need of an interpreter were identified. The questionnaire described in the following sub-section was translated by a professional translation company into the five most common languages in the area (i.e. English, Dari, Arabic, Somali, and Tigrinya). Women who spoke other languages were excluded (n=100), as were those who had moved from the area (n=58) or experienced stillbirth (n=7). In all, 1061 questionnaires were distributed with a pre-paid envelope and an informative leaflet about the study (877 in Swedish and 184 translated). Women consented to participate upon returning the questionnaire, and a reminder was sent to non-respondents after 4–6 weeks.

*Questionnaire*

The questionnaire addressed background characteristics (i.e. age, marital status, country of birth, education level, and parity) and information about pregnancy and childbirth (i.e. gestational length, onset of labor, mode of birth, and self-reported complications). Some items on the questionnaire related to postnatal care, including the time of discharge, women’s opinions about the length of hospital stay, and the model of postnatal care. Women were also instructed to assess their satisfaction with the medical and emotional aspects of the postnatal care received, as well as to provide an overall assessment. Satisfaction with those aspects was rated on 5-point Likert scales ranging from 1 (very satisfied) to 5 (very dissatisfied), which were dichotomized to 1 (very satisfied) and 0 (not very satisfied) in the analysis. These questions about satisfaction have been previously used in studies from Sweden^7,17,18^.

The questionnaire also included the revised version of a previously developed instrument, the Early Postnatal Questionnaire (EPQ)^17^. Consisting of 20 items with three subscales –‘Information’, ‘Postnatal Environment’, and ‘Caring Relationship’ – the EPQ has undergone psychometric evaluation, and the respective Cronbach’s alpha values for the three subscales were 0.876, 0.879, and 0.762, respectively. The first subscale, ‘Information’, addresses common information-related issues experienced by women shortly before discharge, with items concerning physical and emotional health, breastfeeding, pelvic exercise, the resumption of sexual activity, and the care of infants. The second subscale, ‘Postnatal Environment’, addresses possibilities to rest, visiting options, the possibility for partners to stay overnight, opportunities for help with infants, the food provided, and the physical environment. Last, the third subscale, ‘Caring Relationship’, primarily addresses support and encouragement from the staff and being treated with kindness.

### Statistical analysis

The Statistical Package of Social Science version 27.0 was used to analyze the data in a stepwise fashion. Descriptive statistics were used to characterize the participants. Crude odds ratios (OR) with a 95% confidence interval (CI) were calculated as a ratio between the percentages of foreign-born and native-born women for the different categories of the explanatory variables (i.e. background characteristics, length of stay, models of postnatal care, and satisfaction) with medical and emotional aspects as well as the overall assessment of postnatal care. The subscales of the EPQ regarding the content of care (i.e. Information, Postnatal Environment, and Caring Relationship) were investigated. Mean scores and standard deviations for each background variable for the abovementioned areas (i.e. sociodemographic and birth-related background, length of stay, and models of postnatal care) were compared, using analysis of variance (ANOVA)^19^. The subscales were thereafter divided into percentiles, and scores less than the 75th percentile were labelled as indicating ‘low satisfaction’, whereas scores greater than the 75th percentile were labelled ‘high satisfaction’. Multivariate logistic regression analyses adjusting for country of birth were performed to identify which variables mattered the most for being very satisfied with the medical and emotional aspects as well as the overall satisfaction with postnatal care. All statistically significant variables were entered into the model, and the variables that no longer showed statistical significance were removed.

## RESULTS

### Sociodemographic and birth-related background of the participants

In total, 483 of 1061 questionnaires were returned, a response rate of 45.5%. Most women were aged 25–35 years, born in Sweden (85.3%), and living with a partner (96.3%), while 71 women were born in a country outside Sweden. Less than 5% of the women’s pregnancies were premature (4.9%), 16% had their labor induced, and the majority (83%) had a spontaneous vaginal birth. Slightly more women had given birth before (52.1%). The only background variable that differed between women born in Sweden and foreign-born women was civil status, such that foreign-born women were more likely not to live with a partner: 11.3% in foreign-born women and 2.4% in Swedish-born women (OR=5.10; 95% CI: 1.94–13.42) (Table 1).

**Table 1:** Background characteristics in relations to country of birth of postnatal women in Sweden participating in a cross-sectional study in 2017 (N=483)

*Characteristics*	*Women born in Sweden (N=412) n (%)*	*Women born in other countries (N=71) n (%)*	*OR (95% CI)*
**Age** (years)			
<25	44 (10.8)	9 (15.0)	1.55 (0.69–3.34)
25–35 (Ref.)	305 (74.8)	41 (68.3)	1
>35	59 (14.5)	10 (16.7)	1.26 (0.59–2.65)
**Civil status**			
Living with a partner (Ref.)	402 (97.6)	63 (88.7)	1
Not living with a partner	10 (2.4)	8 (11.3)	5.10 (1.94–13.42)***
**Education level**			
Contemporary/high school (Ref.)	212 (52.3)	38 (58.5)	1
College/university	193 (47.0)	27 (41.5)	1.28 (0.75–2.17)
**Parity**			
Primiparas (Ref.)	203 (49.4)	28 (39.4)	1
Multiparas	208 (50.6)	43 (60.6)	1.49 (0.89–2.50)
**Length of pregnancy**			
≥37 completed weeks (Ref.)	377 (95.2)	47 (94.0)	1
<37 weeks	19 (4.8)	3 (6.0)	1.26 (0.36–4.44)
**Onset of labor**			
Spontaneously (Ref.)	328 (84.3)	50 (79.4)	1
Induction	61 (15.7)	13 (20.6)	1.29 (0.71–2.72)
**Mode of birth**			
Vaginal (Ref.)	343 (83.7)	56 (78.9)	1
Instrumental vaginal	21 (5.1)	4 (5.6)	1.16 (0.38–3.52)
Elective cesarean section	21 (5.1)	6 (8.5)	1.75 (0.67–4.52)
Emergency cesarean section	25 (6.1)	5 (7.0)	1.22 (0.45–3.33)
**Complications**			
None (Ref.)	327 (80.0)	59 (84.3)	1
Excessive bleeding	15 (3.7)	2 (2.9)	0.73 (0.16–3.31)
Large rupture	27 (6.6)	2 (2.9)	0.41 (0.09–1.77)
Infection	6 (1.5)	1 (1.4)	0.92 (0.10–7.81)
Other	34 (8.3)	6 (8.6)	0.97 (0.39–2.43)
**Birth experience**			
Very positive (Ref.)	146 (36.8)	29 (43.9)	1
Not very positive	251 (63.2)	37 (56.1)	0.74 (0.43–1.25)

*** p<0.001

### Length of stay and models of postnatal care

Table 2 shows that most respondents went home within 24 h. Whereas 82% regarded the time of discharge as being sufficient, nearly 13% viewed the length of stay as being too short, and 5% viewed the stay as being too long. Most women stayed in a traditional postnatal care ward. Compared with women born in Sweden, women born in another country were likely to be discharged 6–24 h as well as 48 h or more after birth. Moreover, foreign-born women were nearly 4 times more likely to have received co-care in the neonatal ward (OR=3.96; 95% CI: 2.08–7.55).

**Table 2:** Postnatal care in women born in Sweden and women born in other countries

	*Women born in Sweden (N=412) n (%)*	*Women born in other countries (N=71) n (%)*	*OR (95% CI)*
**Time of discharge** (hours)			
6–24	162 (40.8)	34 (51.5)	2.41 (1.20–4.84)*
24–48 (Ref.)	138 (34.8)	12 (18.2)	1
≥48	97 (24.4)	20 (30.3)	2.37 (1.10–5.07)*
**Opinion about length of stay**			
Too short	51 (12.8)	9 (13.4)	1.00 (0.47–2.16)
Sufficient (Ref.)	326 (81.5)	57 (85.1)	1
Too long	23 (5.8)	1 (1.5)	0.24 (0.03–1.87)
**Model of postnatal care^a^**			
Home directly from the labor ward	70 (17.0)	18 (25.4)	1.69 (0.91–3.00)
Traditional postnatal ward	270 (65.5)	42 (59.2)	0.76 (0.45–1.27)
Hotel ward	55 (13.3)	6 (8.5)	0.59 (0.24–1.44)
Postnatal ward and hotel ward	14 (3.4)	4 (5.6)	1.69 (0.54–5.31)
Co-care neonatal ward	34 (8.5)	18 (25.4)	3.96 (2.08–7.55)***

a Several options available. Numbers might not add up to 100% due to internal missing values.

* p=0.05,

** p=0.01,

*** p=0.001.

### Satisfaction with postnatal care

In all, 39.5% of the women reported being very satisfied with the medical aspects of postnatal care, 29.8% with the emotional aspects, and 41.6% with the postnatal care overall. Table 3 shows that there were statistically significant differences between foreign-born and native-born women living in Sweden for two of the areas, namely the medical and the emotional aspects of postnatal care. No between-group differences emerged in the overall assessment of postnatal care.

**Table 3:** Women’s satisfaction with postnatal care in relation to country of birth

	*Women born in Sweden (N=412) n (%)*	*Women born in other countries (N=71) n (%)*	*OR (95% CI)*
**The medical aspects of postnatal care**			
Very satisfied	137 (37.4)	33 (51.66)	1.77 (1.04–3.03)*
Not very satisfied (Ref.)	229 (62.6)	31 (48.4)	1
**The emotional aspects of postnatal care**			
Very satisfied	110 (27.6)	29 (43.3)	2.00 (1.17–3.40)**
Not very satisfied (Ref.)	289 (72.4)	38 (56.7)	1
**The overall assessment of postnatal care**			
Very satisfied	161 (39.8)	36 (52.2)	1.65 (0.99–2.76)
Not very satisfied (Ref.)	244 (60.2)	33 (47.8)	1

* p<0.05,

** p<0.01.

### Background variables in relation to the EPQ’s subscales

*Sociodemographic background and the EPQ*

Women’s sociodemographic background and variables related to postnatal care were analyzed in relation to the subscales of the EPQ (Table 4). Being born in a country outside Sweden was the only sociodemographic variable associated with the EPQ, such that foreign-born women were more likely to score higher than their Swedish-born counterparts.

**Table 4:** The content of postnatal care investigated with the Early Postnatal Questionnaire in relation to women’s background data

*Variables*	*Information*	*Postnatal environment*	*Caring relationship*
	*mean score (SD)*	*mean score (SD)*	*mean score (SD)*
**Age** (years)			
<25	28.75 (10.27)	20.64 (6.01)	21.15 (4.20)
25–35	28.17 (9.46)	19.44 (5.15)	20.69 (4.11)
>35	27.72 (10.87)	19.71 (5.75)	21.44 (3.00)
p	0.849	0.315	0.300
**Country of birth**			
Sweden	27.74 (9.65)	19.23 (5.15)	20.70 (4.02)
Other	30.69 (9.55)	21.87 (6.27)	21.82 (3.51)
p	0.018	0.000	0.029
**Civil status**			
Cohabiting with partner	28.04 (9.73)	19.62 (5.33)	20.88 (3.95)
Single	31.61 (7.90)	19.38 (7.30)	20.55 (4.35)
p	0.126	0.853	0.737
**Education level**			
High school or lower	28.83 (9.94)	19.79 (5.52)	20.73 (4.26)
University	27.61 (9.47)	19.36 (5.28)	20.95 (3.71)
p	0.176	0.380	0.556
**Parity**			
Primiparas	28.25 (9.50)	19.77 (5.53)	20.73 (4.31)
Multiparas	28.11 (9.89)	19.49 (5.30)	21.02 (3.61)
p	0.874	0.579	0.426
**Length of postnatal stay** (hours)			
≤24	27.14 (10.13)	17.95 (5.88)	20.84 (3.81)
25–48	28.70 (9.36)	20.23 (4.75)	20.85 (4.00)
49–72 or longer	28.91 (9.23)	21.38 (4.50)	26.70 (4.31)
p	0.193	<0.001	0.950
**Model of postnatal care**			
Home directly from the labor ward	28.33 (8.33)	16.22 (6.02)	20.55 (3.58)
Traditional postnatal ward	28.72 (9.37)	20.18 (4.61)	21.07 (4.04)
Hotel ward	25.01 (11.01)	18.81 (6.00)	20.09 (4.36)
Postnatal ward and hotel ward	26.37 (10.97)	20.81 (5.00)	19.87 (4.71)
Co-care neonatal ward	28.78 (11.18)	22.53 (5.31)	21.51 (3.36)
p	0.117	<0.001	0.218

*Length of stay and the EPQ*

The actual length of stay was only associated with the subscale ‘Postnatal Environment’, where women who stayed 72 h or longer scored highest (mean=21.38, SD=4.50, p<0.001) but was not statistically significant when comparing foreign-born women and women born in Sweden (Table 4).

*Model of postnatal care and the EPQ*

The model of postnatal care was associated with the ‘Postnatal Environment’ subscale (Table 4). Women who went home directly from the labor ward without staying in the postnatal ward were more likely to score lower (mean=16.22, SD=6.02), as were women who stayed in the hotel ward (mean=18.81, SD=6.00). Women who spent their postnatal stay in the neonatal ward, by contrast, were more likely to score higher (mean=22.53, SD=5.31). Thereafter, the domains were divided by the 75th percentile (i.e. ‘high satisfaction’).

### Multivariate analysis

To determine which variables were most strongly associated with being very satisfied with the various aspects of postnatal care, multivariate regression analyses, adjusted for country of birth, were performed. Table 5 shows that the strongest association, for being very satisfied with all three aspects of postnatal care, was to score above the 75th percentile on the subscale ‘Caring Relationship’, which had AORs ranging from 8.15 to 9.76.

**Table 5:** Most important factors for being very satisfied with postnatal care^a^

*Factors*	*Medical aspects AOR (95% CI)*	*Emotional aspects AOR (95% CI)*	*Overall assessment AOR (95% CI)*
Information^b^	2.30 (1.31–4.03)**	2.05 (1.18–3.56)*	3.81 (2.11–6.89)***
Caring relationship^b^	8.71 (5.05–15.45)***	8.63 (5.04–14.76)***	8.31 (4.83–14.30)***
Postnatal environment^b^		2.69 (1.56–4.63)***	2.04 (1.13–3.69)*
Discharged within 24 hours	0.43 (0.23–0.79)*		

a AOR: adjusted odds ratio; adjusted for country of birth.

b Scoring on the 75th percentile.

* p<0.05,

** p<0.01,

*** p<0.001.

Being very satisfied with the medical aspects of postnatal care was associated with high satisfaction on the ‘Information’ subscale (AOR=2.30; 95% CI: 1.31–4.03), and the subscale ‘Caring Relationship’ (AOR=8.71; 95% CI: 5.05–15.45). Women discharged within 24 hours were less likely to score high on the medical aspects of postnatal care (AOR=0.43; 95% CI: 0.23–0.79).

Similarly, being very satisfied with the emotional aspects was also associated with higher scores on the ‘Information’ subscale (AOR=2.05; 95% CI: 1.18–3.56), ‘Caring Relationship’ subscale (AOR=8.63; 95% CI: 5.04–14.76) and the ‘Postnatal Environment’ subscale (AOR 2.69; 95% CI: 1.56–4.63).

Last, being very satisfied with the overall assessment of postnatal care showed a similar result by scoring high on all three subscales of the EPQ [Information (AOR=3.81; 95% CI 2.11–6.89), Caring Relationship (AOR=8.31; 95% CI: 4.83–14.30), and Postnatal Environment (AOR=2.04; 95% CI: 1.13–3.69)].

## DISCUSSION

The aim of this study was to compare the length and model of postnatal care as well as the content of care between foreign-born and native-born women living in Sweden and to explore factors associated with being very satisfied with various aspects of postnatal care.

The major findings of this study were that foreign-born women were more likely to have a shorter (i.e. <24 h) or longer (i.e. >48 h) length of postnatal stay than women born in Sweden and were 4 times more likely to receive postnatal care in the neonatal ward with their infants. There were no differences in birth outcomes between women born outside Sweden and native-born women. Foreign-born women differed from women born in Sweden in satisfaction with postnatal care by rating the medical and emotional aspects of postnatal care as being higher. Last, after adjusting for country of birth in relation to being very satisfied with the medical and emotional aspects of postnatal care, as well as with the overall assessment, the most important aspect for satisfaction was the content of care, and the subscale ‘Caring Relationship’ outscored all other aspects.

Foreign-born women deviated from Swedish-born women regarding the length of postnatal stay. One explanation for the shorter length of stay might be that they had support from family members at home and preferred to follow their traditions for postnatal care. A similar trend has been highlighted in the meta-synthesis of Benza and Liamputtong^20^ of 15 qualitative studies, which showed that the period of rest was a culturally important practice, as were social support and emotional support, including advice about breastfeeding and caring for newborns. Another explanation might be dissatisfaction with the communication and lack of familiarity with the healthcare system, as shown in a systematic review of 12 studies from five countries^21^. Another systematic review of 51 studies has revealed the financial pressures for many migrant women living in European countries, including difficulties with covering basic living costs, accessing transport to appointments, and paying for costs of essential care^3^, and may also explain the short length of postnatal stays.

The longer length of stay, by contrast, might be explained by more complicated childbirth, which is known to occur more frequently among foreign-born than native-born women living in Sweden^13,16^. A population-based register study has also shown an increased risk of having unplanned and planned cesarean sections for some immigrants compared with women born in Sweden^22^. Usually, cesarean sections demand a longer postnatal stay than vaginal birth does^23^. Foreign-born women were also more likely to receive co-care in the neonatal ward. Although we do not know the reasons for the need for neonatal care, every fourth foreign-born woman was exposed to that model of care despite the non-significant differences in mode of birth and self-reported complications. It is possible that the questionnaire item was simply misunderstood. In Sweden, healthcare is financially subsidized by taxes, and for asylum seekers and undocumented migrants, rights to access healthcare services are covered, and all foreign-born women are offered a free language interpreter if needed. Interpreters thus play a significant role in helping to ensure that childbearing women receive appropriate support and treatment. However, women may not receive that service as much as needed due to a lack of adequate qualified interpreters^24^.

Results regarding satisfaction with postnatal care showed that foreign-born women were also more likely to be very satisfied with the medical and emotional aspects of postnatal care than women born in Sweden were. Those findings contrast past results, including those from the systematic review of Small et al.^21^, in which immigrant women in five countries were less positive about their care than native-born women^21^. One explanation could be that immigrant women compared the care in their home countries versus their host countries and expressed gratitude for the standard of postnatal care that they received^25^. Women’s satisfaction with postnatal care has been related to receiving accurate as well as understandable information. The systematic review of Fair et al.^3^ additionally revealed that a failure to use professional interpreters was a barrier to receiving satisfactory maternity care among migrant women. In short, inadequate professional information sharing with women resulted in misunderstandings. When women’s partners were asked to interpret during postnatal care, women were liable to feel vulnerable and embarrassed and felt that their partners were hesitant to expose their own limited understanding of the host country’s language^3^. As an alternative, cultural doulas in Sweden have described themselves as being personal guides for foreign-born women. They adjusted the support to each woman’s needs and had experience with Sweden’s language, culture, society, and healthcare system that made their work effective^26^.

The most important factors of being very satisfied with the different aspects of early postnatal care were related to the content of care (e.g. Caring Relationships, Postnatal Environment, and Information). Similar aspects have been found to be important in previous studies^3,17,27,28^. Barimani and Vikström^27^ used Haggerty’s typology of continuity of care, in which relational continuity comprises consistency in care or trust in a specific person, whereas informational continuity comprises receiving information related to mothers and infants that empowers parents. In the last category, management continuity, meaning receiving consistent advice, knowing whom to ask, and getting access when needed, seem to comprise postnatal care after discharge. Nevertheless, the aspect of continuity is important for not only the content of postnatal care but also its organization. A randomized controlled trial in Australia, in which 2314 women were randomized to either a caseload midwifery model of care or standard care, showed that women who experienced continuity of care were significantly more likely than their counterparts to be satisfied with the early postnatal care in hospitals, as well as home-based post-partum care^28^. Home-based postpartum care and continuity models of care are prioritized by the World Health Organization^29^ but offered only in a few hospitals in Sweden. In the Stockholm area, that model was introduced in one hospital, where most women went home 6–12 h after birth, 94% received home visits, and 87% reported being very satisfied with the model of care^10^. Given our results, which show that only 41% of the sample were very satisfied with postnatal care, improvements are necessary.

### Strength and limitations

Our study’s observational design limits the opportunity to generalize the findings to a wider population. However, a strength of our study was the translation of the questionnaire into English, Dari, Arabic, Somali, and Tigrinya, which enabled inviting foreign-born women to participate despite often being excluded from research due to language barriers^13,30^. Even so, no questionnaires were returned by the Somali-born population, and foreign-born women were under-represented (i.e. <20% of the sample), which made it impossible to gain the information needed to perform a deeper analysis of non-respondents. Women from Somalia belong to one of the largest groups of immigrants in Sweden and are known to be at high risk of adverse birth outcomes^31^. Lower attendance in antenatal care and worse experiences of maternity care^32^ have been reported for that specific group of women.

Although the response rate of 45.5% was another limitation, a reminder was sent to non-responders. Of them, we gathered information from 47 foreign-born women about how long they have lived in Sweden, which was on average 7.94 years (range: 0–38). It is therefore possible that those women were fairly assimilated into Swedish society.

## CONCLUSIONS

This study adds to the literature on postnatal care and satisfaction among both foreign-born and Swedish-born women. Similar factors, including receiving information, professional care, and a hospital environment that facilitates recovery after labor and birth, explained by our study, have previously been reported internationally; however, this study enables a better understanding of the Swedish context. Further studies are nevertheless needed to assess the model of postnatal care that affords the best opportunities to provide satisfactory care for women and their families regardless of country of origin and language skills. Culturally sensitive and individualized postnatal care with continuity should therefore be prioritized.

## Data Availability

The data supporting this research are available from the authors on reasonable request.
